# Structural Investigation of the Interaction Mechanism between Chlorogenic Acid and AMPA Receptor via In Silico Approaches

**DOI:** 10.3390/molecules27113394

**Published:** 2022-05-25

**Authors:** Wei Zhu, Fengming Wu, Jindie Hu, Wenjing Wang, Jifeng Zhang, Guoqing Guo

**Affiliations:** Department of Anatomy, Neuroscience Laboratory for Cognitive and Developmental Disorders, Medical College of Jinan University, Guangzhou 510630, China; jrwzhu@stu2019.jnu.edu.cn (W.Z.); sj9hwrj@aliyun.com (F.W.); jnuz@163.com (J.H.); wjwang633@163.com (W.W.)

**Keywords:** chlorogenic acid, GluA1, chronic pain, molecular docking, molecular dynamics simulation

## Abstract

Chlorogenic acid (CGA), an important metabolite in natural plant medicines such as honeysuckle and eucommia, has been shown to have potent antinociceptive effects. Nevertheless, the mechanism by which CGA relieves chronic pain remains unclear. α-amino-3-hydroxy-5-methyl-4-isooxazolpropionic acid receptor (AMPAR) is a major ionotropic glutamate receptor that mediates rapid excitatory synaptic transmission and its glutamate ionotropic receptor AMPA type subunit 1 (GluA1) plays a key role in nociceptive transmission. In this study, we used Western blot, surface plasmon resonance (SPR) assay, and the molecular simulation technologies to investigate the mechanism of interaction between CGA and AMPAR to relieve chronic pain. Our results indicate that the protein expression level of GluA1 showed a dependent decrease as the concentration of CGA increased (0, 50, 100, and 200 μM). The SPR assay demonstrates that CGA can directly bind to GluA1 (K_D_ = 496 μM). Furthermore, CGA forms a stable binding interaction with GluA1, which is validated by molecular dynamics (MD) simulation. The binding free energy between CGA and GluA1 is −39.803 ± 14.772 kJ/mol, where van der Waals interaction and electrostatic interaction are the major contributors to the GluA1–CGA binding, and the key residues are identified (Val-32, Glu-33, Ala-36, Glu-37, Leu-48), which play a crucial role in the binding interaction. This study first reveals the structural basis of the stable interaction between CGA and GluA1 to form a binding complex for the relief of chronic pain. The research provides the structural basis to understand the treatment of chronic pain and is valuable to the design of novel drug molecules in the future.

## 1. Introduction

The International Pain Society points out that chronic pain refers to pain that lasts or repeatedly occurs for more than 3 months [1]. The clinical manifestations are usually unpleasant subjective feeling and emotional experience. Chronic pain is one of the most common health problems in today’s society, and seriously affects the physical and mental health of patients. The prevalence rate of chronic pain in China is as high as 31.54%, and 20 million new patients are affected every year [2]. The causes and pathogenesis of chronic pain are complex. It is generally believed that changes in synaptic plasticity caused by central sensitization of the spinal dorsal horn are the basis for the development and maintenance of chronic pain, and changes in the number and location of α-amino-3-hydroxy-5-methyl-4-isooxazolpropionic acid receptors (AMPARs) are the key factors in the development of chronic pain [3]. Glutamate ionotropic receptor AMPA type subunit 1 (GluA1) is the major subunit of AMPAR, and the abnormally high expression of GluA1 is involved in the occurrence of some diseases. GluA1 expression level is regulated by various drug molecules. For example, Corydaline and the systemic administration of rapamycin can treat drug addiction by reducing GluA1 expression, and Desloratadine alleviates allergic rhinitis by downregulating GluA1 expression [4,5,6]. Studies have shown that chronic pain can increase the expression level of GluA1, leading to more active synaptic transmission and increased cell excitability, and specific blocking of GluA1 can produce significant analgesic effect [7,8]. Currently, the drugs for the treatment of chronic pain mainly include non-opioid analgesics and opioids, but long-term use of these drugs has obvious side effects and poor therapeutic effects [9,10].

In the process of modern pharmaceutical research and development, the application of natural plant drugs has attracted much attention. Chlorogenic acid (CGA) is a phenolic acid generated by caffeic acid and quinic acid, which has a variety of biological functions, including antioxidant, antibacterial, anti-inflammatory, and neuroprotective activity [11]. It is usually found in plants, fruits, and vegetables, such as honeysuckle, eucommia, eggplants, and coffee beans [12,13,14]. These CGA-rich plants are commonly used in traditional medicine for relieving pain [15]. At the same time, researchers have found that CGA can relieve chronic pain in animal models, such as inflammatory pain and chronic neuropathic pain caused by formalin and carrageenan [16]. Recent studies indicated that CGA could specifically inhibit synaptic expression of GluA1 subunit, reduce excitatory toxicity, and relieve chronic pain [10,17,18]. Nevertheless, there are few reports on how CGA acts on GluA1 to exert an analgesic effect [16,18].

In this study, Western blot, surface plasmon resonance (SPR) assay, molecular docking, molecular dynamics (MD) simulation, and binding free energy calculation and decomposition were employed to explore the binding mode and dynamic interaction between GluA1 and CGA. By means of computer simulation methods, the mechanism of CGA in the treatment of chronic pain is revealed at the atomic level, which provides a theoretical basis for the design and development of new drugs.

## 2. Materials and Methods

The hardware platform used in the computational simulations in this study was based on a dual-channel Intel Xeon E5-2696V4 server, and the operating system was Ubuntu 20.04 LTS.

### 2.1. Reagents and Cell

CGA (3- caffeoylquinic acid, (1S,3R,4R,5R)-3-[(E)-3-(3,4-dihydroxyphenyl) prop-2-enoyl]oxy-1,4,5-trihydroxycyclohexane-1-carboxylic acid) was purchased from Sigma-Aldrich (C3878, St. Louis, MO, USA). Recombinant human GluA1 (AMPA subtype) protein was purchased from Abcam (ab112297, Cambridge, UK). The mouse dorsal root ganglion (DRG) cells purchased from Procell (CP-M166, Wuhan, China) were cultured in a medium consisting of Neurobasal-A medium (GIBCO, 21103049) with 2% B-27 (GIBCO, 17504044) at 37 °C in a 5% CO_2_ humidified incubator. Half of the culture medium was replaced every 3 days.

### 2.2. Western Blot Analysis

BCA protein assay kit (Beyotime) was used to measure protein concentrations. The protein samples were separated by SDS-PAGE and then transferred to a PVDF membrane (Millipore, MA, USA). The primary antibodies used in this study were as follows: GluA1 (Sigma-Aldrich, ABN241; 1:1000) and β-actin (Servicebio, Gb12001; 1:3000). The protein bands were visualized using enhanced chemiluminescence reagents (ThermoFisher, Rockford, MI, USA). We analyzed the difference in the inhibitory effect of different concentrations of CGA on GluA1 expression by comparing it with the control group. Image J software was used for the determination of the density of the bands.

### 2.3. SPR Assay

To verify whether CGA directly binds to GluA1, we performed an SPR experiment using Biacore T200 instruments (Cytiva, Fremont, CA, USA). We pretreated a CM5 sensor chip with running buffer (1 × PBS, 0.05% Tween-20, pH = 7.4). The sensor chip was activated by an amine coupling kit (Cytiva, Fremont, CA, USA) and GluA1 was immobilized on the sensor chip. The serial concentrations of CGA (6 × 10^−5^–3.84 × 10^−3^ M) were injected into the flow system separately at a flow rate of 30 μL/min. The association time was 60 s and the dissociate time was 60 s. We analyzed the equilibrium constant (K_D_) and plot steady-state fit curve using the Biacore T200 Evaluation software (Cytiva, Fremont, CA, USA).

### 2.4. Molecular Docking

#### 2.4.1. Structure Preparation

In this study, we selected the ligand binding domain (LBD) of GluA1 as the receptor protein. The LBD structure of GluA1 is composed of the S1 and S2 fragments, which are linked to M1 and M2, respectively, as well as the M3 fragment of the transmembrane region. The N-terminal region of S1 forms a short linker with the C-terminal region of the protein extracellular N-terminal domain, and the S2 fragment forms a closed structure with M3 and M4 [19] (Supplementary Figure S1A). The structure file (PDB ID: 7OCD, 3.50 Å) was obtained from the RCSB protein data bank database (www.rcsb.org) [20]. The structure visualization software PyMOL v2.3.4 was used to remove irrelevant structures, crystal waters, and co-crystal ligands in the original conformational file (Supplement Figure S1B). In this study, ChemBioDraw Ultra v12 was used to draw the structure of ligand CGA, and ChemBio3D Ultra v12 was employed to optimize its structure in MM2 molecular mechanics (Supplement Figure S2). Then, we input the receptor and ligand structure files into AutoDockTools v1.5.6 to generate the “pdbqt” format files before docking simulation.

#### 2.4.2. Molecular Docking Simulation of GluA1 and CGA

We used AutoDockTools v1.5.6 for global docking and to set the search space size and coordinates. The central coordinates were (13.746, 4.621, 14.997), the grid spacing was 1 Å, and grid number was 50 × 50 × 50. AutoDock Vina v1.1.2 was used to complete the semi-flexible molecular docking simulation between GluA1 and CGA, in which the conformation of ligand CGA remained flexible, while the structure of the receptor protein was set to be rigid during the docking process. The Broyden–Fletcher–Goldfarb–Shanno (BFGS) algorithm was used to search for the optimal binding conformation [21], the number of binding conformation outputs was 20, and the energy range of conformation search was set to 5 kcal/mol. After the construction of configuration files, we input the corresponding commands to perform the docking simulation and checked the results of the molecular docking using VMD v1.9.4a53.

### 2.5. MD Simulation

To understand in-depth the dynamic interaction between GluA1 and CGA and the stability of the interaction, we used the open-source software GROMACS v2019.3 to conduct 90 ns MD simulation for the optimal binding conformation [22]. First, we used the pdb2gmx module and selected the GROMOS54A7_atb force field—the force field suitable for the protein nucleic acid systems and containing non-standard atomic types—to generate the receptor protein topology file and its location restriction file. Meanwhile, we browsed the online database ATB (Auto Topology Builder http://atb.uq.edu.au, accessed on 14 October 2021) to obtain the corresponding parameter file of CGA [23]. Then, we set the box of the system as the hexahedron type and placed the binding complex in the central position of the system. The minimum distance from the edge was 1 Å. A single-point charge water model molecule was selected as the solvation molecule of the simulated system. As there was no net charge in the biosystem, we added 3 Cl^−^ ions to substituted water molecules to antagonize the charges in the initial system to ensure the electrical neutrality. Finally, the simulation system contained a GluA1–CGA complex structure, water molecules, and antagonistic ions with a total of 68,471 atoms. Before the production run, we used the steepest descent algorithm to minimize the energy of the initial system in 50,000 steps, and the energy convergence value was 100 kJ (mol·nm)^−1^, so as to eliminate the potential conformational structural clashes in the initial system. Then, we performed 100 ps canonical ensemble (the constant-temperature, constant-volume ensemble, NVT) equilibrium and 200 ps constant pressure/temperature (the constant-temperature, constant-pressure ensemble, NPT) equilibrium for the system. After the equilibrium system was balanced, the atom position restriction of the system was released and the production phase dynamics simulation of 90 ns was performed. In this stage, the particle mesh Ewald algorithm was used to deal with the long-range electrostatic interaction between molecules. The Coulomb cut-off value was set at 1.2 nm, and the spacing of the Fourier transform grid points was 0.16. In addition, the cutoff value of the short-range van der Waals interaction was set to 1.2 nm. The v-rescale temperature controller with an improved Berendsen method was used to control the temperature of the system, and the Parrinello–Rahman method was used to control the pressure of the system.

### 2.6. MMPBSA Binding Free Energy Calculation and Decomposition

The trajectory of last 10 ns of the stable state of the system was sampled and analyzed, and the trajectory snapshot was extracted every 100 ps. The molecular mechanics/Poisson-Bolzmann surface area (MMPBSA) method was applied to calculate the binding free energy between GluA1 and CGA ($∆G_{binding}$). The formulas were as follows:

$$∆G_{binding}=∆G_{complex}-\left(∆G_{GluA1}+∆G_{CGA}\right)=∆E_{MM}+∆G_{solv}-TS$$

$$∆E_{MM}=∆E_{int}+∆E_{vdw}+∆E_{ele}$$

$$∆G_{solv}=∆G_{PB}+∆G_{SA}$$

where $∆G_{complex}$ represents the total Gibbs free energy of the GluA1–CGA complex and $∆G_{GluA1}$ and $∆G_{CGA}$ represent the free energy of GluA1 and CGA in solution, respectively. The free energy of each composition is composed of $∆E_{MM}$ $∆G_{solv}$, and TS; $∆E_{MM}$ represents the molecular mechanics potential energy in a vacuum, and this energy item includes the internal bond energy ($∆E_{int}$), the van der Waals interaction ($∆E_{vdw}$), and electrostatic interaction ($∆E_{ele}$); and $∆G_{solv}$ represents the solvation free energy, including the polar energy contribution ($∆G_{PB}$) and nonpolar energy contribution ($∆G_{SA}$). In addition, TS represents the contribution of the conformational entropy change of the system at a constant temperature (*T*). Owing to the low accuracy of normal mode analysis, significant errors will be introduced into the results, which results in the uncertainty of the results; meanwhile, the computational cost is very expensive, so we usually ignore the structural entropy contribution of the system when calculating the binding free energy [24,25].

### 2.7. Statistical Analysis

The experimental data were expressed as mean ± SD, and GraphPad Prism 9 software was used to create graphs and statistics. Statistical analysis was performed with one-way ANOVA followed by Bonferroni-corrected post-hoc tests. *p* < 0.05 was considered statistically significant.

## 3. Results

### 3.1. Effect of CGA on GluA1 in DRG Cells

**Figure 1:**
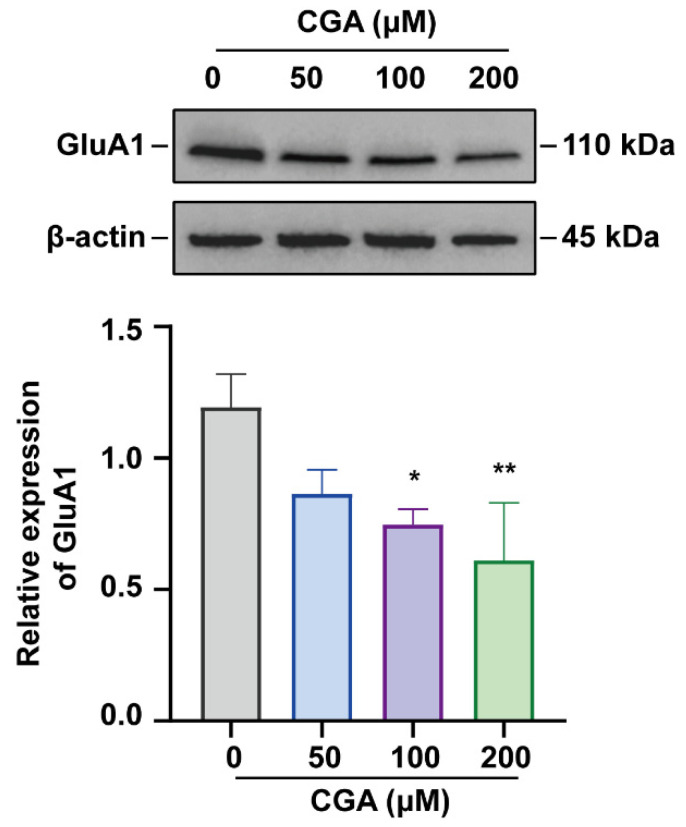
Effect of CGA on GluA1 in dorsal root ganglion (DRG) cells. The protein expression levels of GluA1 were assayed by Western blot analysis. n = 3, β-actin expression was used as loading control. * *p* < 0.05, ** *p* < 0.01, as compared with the control group (0 μM).

We explored the changes in the protein expression levels of GluA1 of DRG cells treated with different concentrations of CGA for 72 h. The results showed that, as the concentration of CGA increased (0, 50, 100, and 200 μM), the expression level of GluA1 was significantly decreased (Figure 1). The Western blot analysis demonstrated that CGA could inhibit the expression of GluA1, which was in accordance with the previous studies [10,18].

### 3.2. CGA Directly Binds to Human GluA1

**Figure 2:**
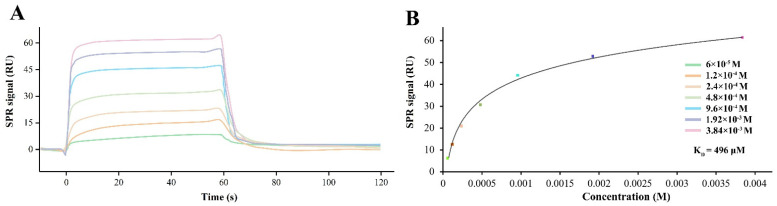
Surface plasmon resonance (SPR) analysis of CGA binding to GluA1. (**A**) The sensor chip plot indicted that the binding of CGA to GluA1 led to the refractive angle shift, which induced a change in the value of the response signal. (**B**) The steady-state binding curve.

With different concentrations of CGA flowing across the sensor chip surface immobilized with GluA1, the direct binding of CGA to GluA1 led to alterations to the response signal (Figure 2A). As the drug concentration increased, the response signal showed a significant increase, which indicated that CGA could bind directly to GluA1 in a dose-dependent manner. The K_D_ of the CGA–GluA1 interaction was calculated as 496 μM using a steady-state fit model (Figure 2B).

### 3.3. Molecular Docking Study

**Figure 3:**
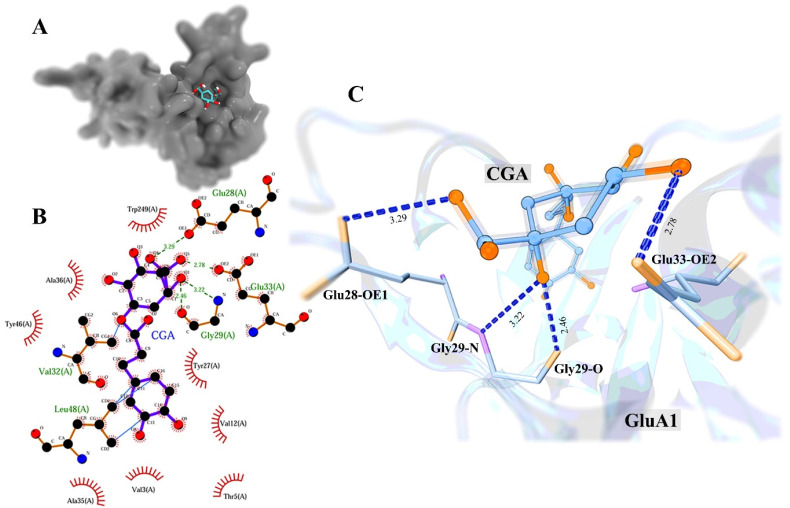
The analysis of binding conformation of GluA1 and CGA. (**A**) The binding pose of GluA1 and CGA; (**B**) 2D diagram of the interaction between CGA and GluA1, where green dotted lines represent the intermolecular hydrogen bonds, red spoked arcs represent the hydrophobic interactions, and light blue lines represent the external bonds; (**C**) the structural details of hydrogen bonds between GluA1 and CGA in 3D view.

Because the interaction between ligand and protein is a thermodynamic equilibrium process, a stable binding conformation usually has a low affinity score. In this study, the lowest binding affinity score of the binding complex was −6.8 kcal/mol, and the corresponding structure is shown in Figure 3A. We found that GluA1 and CGA interacted mainly through intermolecular hydrogen bonding, hydrophobic interaction, and external bonds with receptor protein (Figure 3B,C). For details of the binding mode analysis, please see the Supplementary Material.

### 3.4. The Analysis of GluA1–CGA Complex MD Simulation

**Figure 4:**
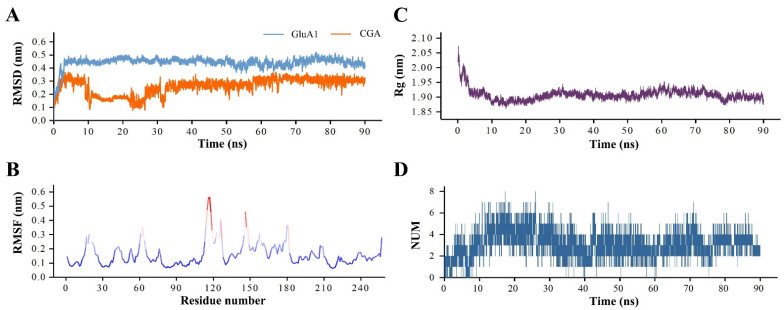
The analysis of molecular dynamics (MD) simulation. (**A**) Root mean square deviation (RMSD) curves of GluA1 (blue) and CGA (orange); (**B**) root mean square fluctuation (RMSF) plot of the receptor GluA1 in MD simulation; (**C**) radius of gyrate (Rg) plot of backbone of the receptor GluA1 in MD simulation; (**D**) hydrogen bond plot of CGA with GluA1 in MD simulation.

MD simulation could provide valuable information to understand the dynamic properties of protein–ligand interactions [26]. In this study, we selected the optimal binding conformation for 90 ns MD simulation to observe the interaction between GluA1 and CGA and structural stability of the complex on the time scale. First, we calculated the root mean square deviation (RMSD) of GluA1 and CGA by fitting the initial structure with the least square method to characterize the stability of receptor and ligand structures during the simulation (Figure 4A). The RMSD of the receptor protein was relatively stable overall during the MD simulations, fluctuating steadily at 0.45 nm. In the initial period of the simulation (0–5 ns), the RMSD of GluA1 showed a rapid upward trend. We speculated that this was because, in previous molecular docking, we set the structure of the receptor protein to be rigid. Therefore, after releasing the position restriction in the MD simulation, the structure of the receptor would experience a temporary adjustment. Meanwhile, we also calculated the RMSD of the ligand CGA and found that, although there were some fluctuations from 0 to 33 ns at the beginning of the simulation, the structure of the ligand CGA reached a stable state when the MD simulation came near 33 ns, and the RMSD value remained at the level of 0.28 nm (33–90 ns). We considered the fluctuation of ligand RMSD at the beginning of the simulation as a process of mutual adaptation of CGA and GluA1, which helped to fit together.

We calculated the root mean square fluctuation (RMSF) of the backbone atoms of GluA1 to analyze the structural flexibility of each residue during the simulation (Figure 4B). The regions with high flexibility were mainly concentrated in the 15–23, 60–77, 115–130, and 144–182 residue segments, and the corresponding conformations were distributed in the loop regions of side chain and structural connections. On the other hand, segments 27–38, 79–109, and 214–249 showed low flexibility. The structure of residues in the binding region was relatively stable during the simulation, which was conducive to the interaction between GluA1 and CGA.

In this study, we also calculated the radius of gyrate (Rg) of receptor GluA1 to observe the folding dynamic behavior of the protein structure in binding with CGA (Figure 4C). During 0–10 ns, the Rg value decreased rapidly from 2.02 nm to 1.88 nm, indicating that the GluA1 structure showed continuous folding behavior during the initial 10 ns of the simulation. During the simulation from 10 ns to 30 ns, the Rg value slowly increased to 1.90 nm, and the GluA1 structure was slightly extended. After that, until the end of the simulation, although the Rg value was slightly adjusted at 78 ns, the overall structure did not change significantly, and the Rg value fluctuated steadily at the level of 1.90 nm.

The hydrogen bond interaction plays an important role in the receptor–ligand molecular interaction. In this study, we analyzed the time distribution characteristics of the hydrogen bonds between GluA1 and CGA by calculating the number of intermolecular hydrogen bonds during MD simulation (Figure 4D). During the 90 ns MD simulation, the number of hydrogen bonds between GluA1 and CGA ranged from 0 to 8, and the number of hydrogen bonds increased to 8 in the period of 0–15 ns. From then on to 30 ns, the number of hydrogen bonds was mainly concentrated in 3–6. During 30–40 ns, the number of intermolecular hydrogen bonds decreased to 2–4. Subsequently, the number of intermolecular hydrogen bonds remained stable at 2–4 until the end of the simulation. The dynamic change in the number of hydrogen bonds might be due to the movement of the atomic position, which exposed potential hydrogen bond interaction sites in the simulation period, resulting in the increase or decrease in the number of hydrogen bonds.

### 3.5. Binding Free Energy Calculation and Decomposition

In this study, the equilibrium trajectory of the last 10 ns of MD simulation was saved and extracted every 10 frames to calculate the binding free energy of GluA1 and CGA. The results showed that the binding free energy of GluA1–CGA was −39.803 ± 14.772 kJ/mol, and the free energies of van der Waals, electrostatic, and non-polar solvation were all negative, indicating that these interactions could prompt the binding between GluA1 and CGA, and that van der Waals and electrostatic interaction played a dominant role of binding. However, the polar solvation energy value was greater than zero, which was not conducive to the combination (Table 1).

**Figure 5:**
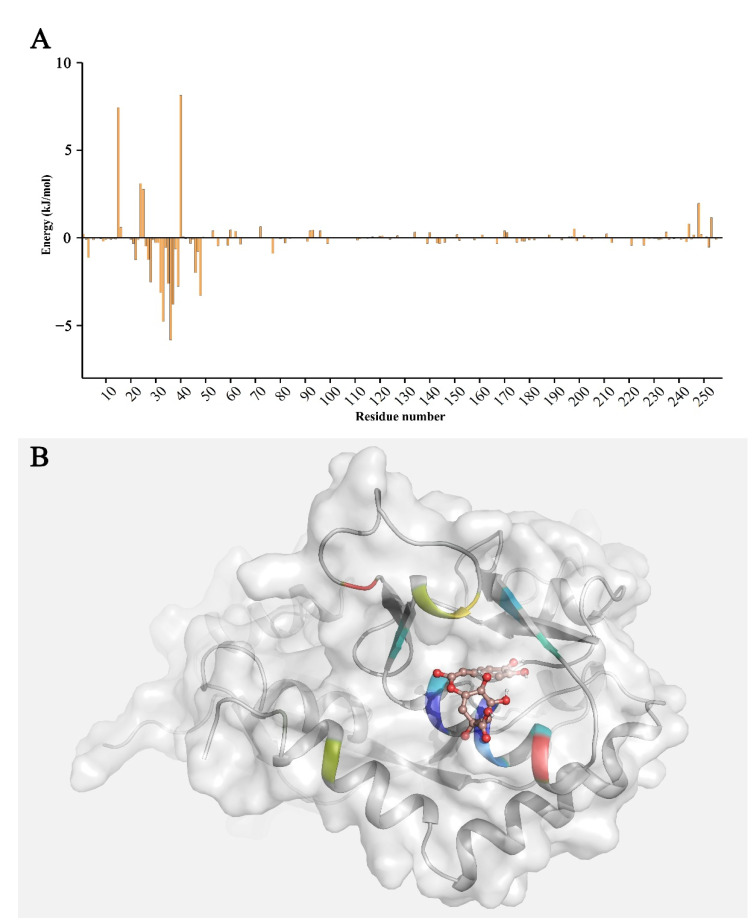
The distribution and structural visualization of per-residue binding free energy. (**A**) The bar graph represents the binding free energy contribution of each amino acid; (**B**) visualization of the binding free energy between CGA and GluA1, where the color of the residues closer to dark blue indicates the higher energy contribution of the residue, and the color of the residues closer to red indicates the lower energy contribution of the residue.

We decomposed the binding free energy to analyze the energy contribution of a single amino acid to the interaction between GluA1 and CGA (Figure 5A). Residues 26–39 and 44–48 in GluA1 promoted the binding of CGA and GluA1, which confirmed the accuracy of molecular docking. The residues with binding energies less than −3 kJ/mol included Val-32, Glu-33, Ala-36, Glu-37, and Leu-48, where the binding energy was −3.1225 kJ/mol, −4.7781 kJ/mol, −5.8385 kJ/mol, −3.7967 kJ/mol, and −3.2973 kJ/mol, respectively. These key residues might be the basis of the binding between GluA1 and CGA. At the same time, the binding energies of Lys-15, Asn-24, Asp-25, Lys-40, and Lys-248 were greater than zero, which did not contribute to the interaction. In order to intuitively observe the energy contribution of each residue, we mapped the energy data into the 3D structure of receptor GluA1 (Figure 5B). The favorable residues for binding were mainly concentrated at the bottom of the binding pocket, surrounding the ligand CGA, while the unfavorable residues were located outside the binding pocket.

## 4. Discussion

In this study, we found that, when the DRG cells were treated with CGA, GluA1 showed a significant decrease in protein expression. After that, the SPR assay result indicated that CGA directly binds to human GluA1. The interaction of GluA1 with CGA in this study belongs to the type of small molecule–protein interaction and the binding strength is comparable to other similar compounds, where the K_D_ values are at the same order of magnitude, such as studies of the interaction of natural polyphenol Honokiol with α-Hemolysin (K_D_ = 172 μM) and Tuftsin with human ACE2 (K_D_ = 460 μM) [27,28]. In addition, we applied the molecular docking to construct the GluA1–CGA binding complex model, and analyzed the binding mode of GluA1 and CGA, where the binding of CGA to GluA1 was mainly through intermolecular hydrogen bond, hydrophobic interaction, and external bonds. Subsequently, through the MD simulation analysis, we found that the GluA1–CGA binding complex was stable, and the residues around the binding region showed low structural flexibility. Then, the dynamic trajectory was sampled to analyze the binding free energy, and the intermolecular binding free energy of GluA1 and CGA was calculated to be −39.803 ± 14.772 kJ/mol, in which van der Waals interaction and electrostatic interaction were the main driving forces to promote the stable binding. The key residues with significant energy contributions were identified by decomposing the binding energy to the level of a single amino acid.

AMPAR is an important receptor mediating rapid excitatory synaptic transmission in vivo and plays an important role in the regulation of synaptic plasticity in physiological or pathological conditions. GluA1, a key subunit of AMPAR, is critical in the regulation of synaptic plasticity, neuronal development, and various neurological disorders [29]. It has been reported that GluA1 deficiency enhances long-term spatial memory to some extent, as well as affects short-term spatial working memory. Studies have found that GluA1-mediated synaptic plasticity plays an important role in the early development of Alzheimer’s disease [30,31]. In addition, drug addiction also increases GluA1 protein expression to promote the rewarding effects of drugs, such as opioid analgesics [32]. Nowadays, pain and its associated diseases remain a problem that plagues human society. Even though various plants are widely used in pain treatment, especially in the South African population, the related ethnomedicinal understanding and the anti-inflammatory and analgesic mechanisms of medicinal plants are poorly known and need to be further investigated [33,34,35]. It is generally believed that the main mechanism is high expression of GluA1, which leads to abnormal excitation of AMPAR and the enhancement of peripheral and central sensitization, while inhibition or loss of GluA1 can relieve hypersensitivity to pain [36]. There is still a lack of effective measures to treat the chronic pain. As natural products can provide a unique chemical structural diversity, the pharmacological exploration of medicinal plants has become one of the efforts of the pharmaceutical industry. Natural herbal medicines with analgesic properties have become the basis for the development of new analgesics [37]. CGA has a variety of biological activities, including anti-inflammatory, analgesic, and neuroprotection activities [38,39]. Previous experiments have shown that CGA can significantly relieve chronic pain by inhibiting GluA1 and affecting synaptic plasticity [40]. However, the molecular mechanism by which CGA acts on GluA1 remains elusive and further research is needed. Therefore, it is of certain value to explore the interaction between CGA and GluA1 using molecular simulation methods.

In this study, molecular docking was used to identify the binding pose between CGA and GluA1. Through the analysis of the interaction mode, it was found that four hydrogen bonds could be formed between CGA and GluA1, of which the length of the hydrogen bond with Glu-33 was the shortest (2.78 Å), and the bond energy was stronger than that of other hydrogen bonds. Val-3, Thr-5, Val-12, Tyr-27, Ala-35, Ala-36, Tyr-46, and Trp-249 in the receptor were residues with a hydrophobic contribution. Meanwhile, we found external bonding interactions in the binding conformation. In order to deeply understand the dynamic interaction between CGA and GluA1 in organisms, MD simulation was used to verify the binding stability and interaction details of their binding, as well as analysis of the binding free energy to obtain the key residue sites: Val-32, Glu-33, Ala-36, Glu-37, and Leu-48. These key residues may be the basis for the stable interaction between CGA and GluA1 to play a pharmacology role.

In conclusion, this study confirmed that the use of CGA was able to decrease GluA1 protein expression at the cellular level, and demonstrated the direct interaction between the two molecules using the biosensor chip. Further, we used molecular simulation techniques to reveal the stable interaction between CGA and GluA1 and to identify the key residues at the atomic level. We hope this study will provide valuable insights into analgesic drug design.

## Figures and Tables

**Table 1:** Binding free energy between CGA and GluA1. Each energy value is given in the form of “mean ± standard deviation”.

Energy Item	Energy (kJ/mol)
$∆E_{vdw}$	−95.763 ± 13.707
$∆E_{ele}$	−63.083 ± 9.975
$∆G_{PB}$	132.238 ± 16.630
$∆G_{SA}$	−13.194 ± 1.277
$∆G_{binding}$	−39.803 ± 14.772

## Data Availability

The data associated with the study are available with the corresponding author and can be produced upon reasonable request.

## Supplementary Materials

The following supporting information can be downloaded at: https://www.mdpi.com/article/10.3390/molecules27113394/s1, Figure S1: The structure preparation of the receptor protein; Figure S2: Planar and stereo structures of ligand CGA.
