# Supplementary material for: Structural Investigation of the Interaction Mechanism between Chlorogenic Acid and AMPA Receptor via In Silico Approaches
Source: Molecules. 2022 May 25;27(11):3394. doi: 10.3390/molecules27113394 (PMC9182211; doi:10.3390/molecules27113394)
Supplement: Supplementary file 1 [file molecules-27-03394-s001.zip › molecules-1712146-supplementary.pdf]

Supplementary Material

Detailed binding mode analysis

Among the hydrogen bonding interaction, the OE1 atom of the side chain of γ-carboxylic acid in Glu-28 formed a hydrogen bond of 3.29 Å to the O4 atom of CGA. The O atom and N atom of residue Gly-29 formed hydrogen bonds of 2.46 Å and 3.22 Å respectively with the O1 atom of CGA, the OE2 atom of Glu-33 formed a hydrogen bond of 2.78 Å with the O5 atom of CGA. The residues involved in hydrophobic interaction were Val-3, Thr-5, Val-12, Tyr-27, Ala-35, Ala-36, Tyr-46, and Trp-249. In addition, 4 external bonds could be formed between CGA and GluA1 (Leu-48, Val-27).

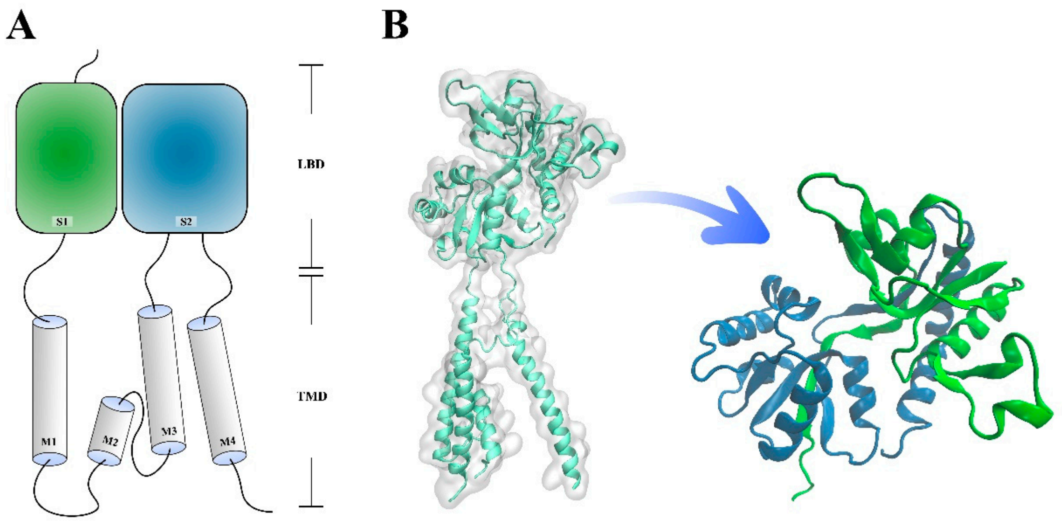

Supplement Fig. 1 The structure preparation of the receptor protein. (A) Schematic of the GluA1 subunit; (B) The structure of receptor GluA1 before and after pretreatment.

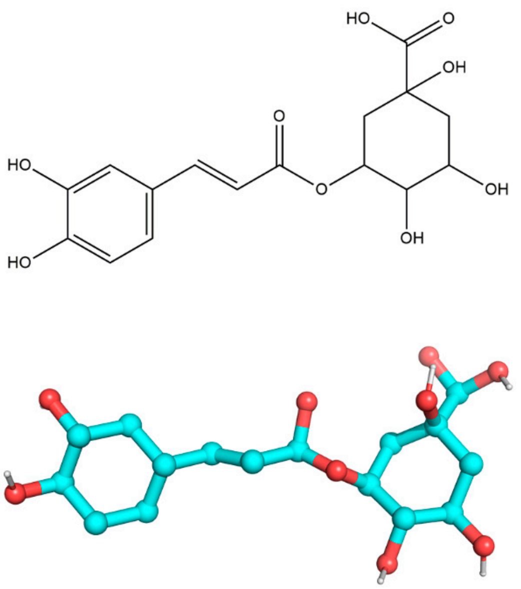

Supplement Fig. 2 Planar and stereo structures of ligand CGA
